# Inclusion of Yerba Mate (*Ilex paraguariensis*) Extract in the Diet of Growing Lambs: Effects on Blood Parameters, Animal Performance, and Carcass Traits

**DOI:** 10.3390/ani10060961

**Published:** 2020-06-01

**Authors:** Richard R. Lobo, Rafaela Vincenzi, Danny A. Rojas-Moreno, Annelise A. G. Lobo, Caroline M. da Silva, Vanderlei Benetel-Junior, Laura R. Ghussn, Vinícius C. Mufalo, Alexandre Berndt, Sarita B. Gallo, Rafael S. B. Pinheiro, Ives C. da S. Bueno, Antonio P. Faciola

**Affiliations:** 1 College of Animal Science and Food Engineering, University of São Paulo, Pirassununga 13635-900, SP, Brazil; vincenzi.rafaela@gmail.com (R.V.); dannymoreno.zoot@gmail.com (D.A.R.-M.); anneliselobo@usp.br (A.A.G.L.); karolmsp.2@gmail.com (C.M.d.S.); vanderlei.benetel@usp.br (V.B.-J.); lauraghussn@usp.br (L.R.G.); viniciusmufalo@usp.br (V.C.M.); saritabgallo@usp.br (S.B.G.); ivesbueno@usp.br (I.C.d.S.B.); 2 Department of Animal Sciences, University of Florida, Gainesville, FL 32608, USA; afaciola@ufl.edu; 3 Embrapa Southeast Livestock, Brazilian Agricultural Research Corporation, São Carlos 13560-970, SP, Brazil; alexandre.berndt@embrapa.br; 4 College of Engineering, São Paulo State University, Ilha Solteira 15385-000, SP, Brazil; rafael.pinheiro@unesp.br

**Keywords:** additive, performance, plant extract, small ruminants, white blood cells

## Abstract

**Simple Summary:**

Yerba mate (*Ilex paraguariensis*) is a plant widely used for human consumption in Latin America, with important health benefits for people. However, limited knowledge on its effects on animal health and nutrition are available. In this study, we evaluated the inclusion of yerba mate extract at levels up to 4% of the dry matter in the diets of growing lambs and its effects on blood parameters, animal performance, body metrics and carcass traits. Yerba mate extract up to 2% of inclusion had positive effects on feed intake and animal performance; however, greater inclusion rates had negative effects on feed intake and animal performance. Supplementation of yerba mate extract up to 2% also increased white blood cells and globulins, which have been closely related to a better immune response in animals.

**Abstract:**

This study aimed to evaluate levels of yerba mate (*Ilex paraguariensis*) extract (YME), as a feed additive in the diets of growing lambs on serum biochemical parameters and hematological indices, animal performance, body metrics and carcass traits. Thirty-six entire (nine per treatment), male growing lambs, weighing 23.8 ± 3.7 kg, were fed the experimental diets which were treatments consisting of increasing levels of YME (0, 1, 2, and 4% inclusion on a dry matter [DM] basis) during an experimental period of 53 days. The experiment was carried out in a randomized block design, which initial body weight was used as blocking factor and the results were analyzed by orthogonal contrasts (linear, quadratic, and cubic). Yerba mate extract did not change the general health status of the animals; however, inclusions of up to 2% of the extract increased globulins (*p* = 0.05) and white blood cell count, as segmented neutrophils (*p* = 0.02) and lymphocytes (*p* = 0.04). Additionally, inclusion of up to 2% YME increased dry matter intake, final weight gain, total and daily gain (*p* < 0.05), also tended to increase ribeye area and reduce fat thickness (*p* < 0.10); however, YME above 2% of inclusion reduced animal productive parameters (*p* < 0.05). In conclusion, levels up to 2% of YME were beneficial to the health and productive parameters of growing lambs.

## 1. Introduction

Nutritionally and physiologically, weaning is a challenging period for lambs. At a few months of age lambs are separated from the ewe, causing a great amount of stress to the animal, with potential negative effects on the gastrointestinal mucosa, and consequently suppression of the immune system and reduced growth performance [1]. In addition, the health of the animals is one of the most important features that influences productivity parameters. A high correlation between health status and nutritional balance was described by Goff [2]; therefore, it is important to evaluate nutritional strategies to prevent disorders during this stage of life.

One of the strategies most used by researchers and producers worldwide is the use of feed additives to improve animal health and production during the post weaning period [3,4,5,6]. However, some synthetic feed additives are prohibited by the European Union [7] and the United States [8], because of the potential anti-microbial resistance and possible impact on human health. These regulations open a wide niche for researchers to evaluate alternative products such as probiotics, prebiotics, enzymes, organic acids, organic minerals, plant extracts, essential oils, and others that could have a positive impact on health and production of animals.

Several plants and their extracts can be used as feed supplements [9,10,11], and the leaves of yerba mate (*Ilex paraguariensis*) is a typical product used for human nutrition in South American countries. Its bioactive compounds are mainly composed by phenolic compounds [12,13,14], alkaloids [15,16] and saponins [17,18,19,20]. A review on the functions of yerba mate in human nutrition has been described, including its antioxidant, antimicrobial, anti-obesity, anti-inflammatory, and anti-diabetic properties [21,22,23].

Animal studies using yerba mate are very limited, and some have reported positive results. The inclusion of yerba mate extract in dairy animals diet, ewes supplemented with dried leaves at doses of 0 and 2.5% (DM basis) and cows supplemented with dried leaves at 0, 250, 500, and 750 g/day/animal, were reported to have positive impacts on milk yield and composition [24,25]. Also, on meat quality of beef cattle [26] fed dried plant extract (0, 0.5, 1, and 1.5% of DM), greater antioxidant activity of meat and milk were also reported [25,26].

However, more studies are needed to better understand the mechanisms of action of yerba mate compounds and their effects during challenging stages of animal lifecycle, including weaning. In this context, our hypothesis was that increasing levels of yerba mate extract in the diet of weaned lambs would improve their performance and health status. The objectives were to evaluate the performance parameters, body and carcass yield and characteristics and to determine the health status by assessing blood counts and biochemical analyses of weaned lambs fed increasing levels of dietary yerba mate extract.

## 2. Material and Methods

All procedures using animals were approved by the Institutional Animal Care and Use Committee (protocol number CEUA 3497040618) of the College of Animal Science and Food Engineering from the University of São Paulo (FZEA-USP), Brazil.

### 2.1. Location, Animals and Experimental Design

Animals were born and raised until weaning on a commercial farm and had access to pasture and feed supplementation on a creep-feeding system. After weaning, they were transported to the experimental unit of FZEA-USP, where the experiments were carried out, from 26 June to 14 August 2018. Average temperature and humidity for the period were 17.9 °C and 53.7%, respectively (data obtained from FZEA-USP weather station). The facility in which the animals were allocated was composed of individual pens (width = 75 cm and length = 115 cm) with elevated plastic floor (50 cm from ground) with holes to help removing the urine and feces to keep the pen clean.

Thirty-six entire male lambs from the same herd (crossbred [Texel × Santa Inês] × Dorper) weighing 23.77 ± 3.70 kg at age of 90 days, were used in this trial. Upon arrival at the experimental facilities, all animals were dewormed with levamisole hydrochloride, following the dose recommendations from the manufacturer. The animals were weighed and assigned into nine blocks. Each block had four animals with similar weight, and those four animals within each group were randomly assigned to one of four experimental treatments, according in a randomized block design. Each animal was placed into an individual pen with individual water and feeder access and fed the control diet (0% of yerba mate extract as presented in Table 1).

### 2.2. Diet and Experimental Treatments

Animals were fed daily at 08:00 h and 16:00 h, and diets were formulated according to the nutritional requirements for finishing lambs [27]. In the basal diet forage:concentrate ratio was composed of 40% corn silage and 60% concentrate (in a dry matter basis) while the concentrate was a mixture of ground corn, soybean meal, salt, dicalcic phosphate, mineral premix, and kaolin and/or yerba mate extract. Kaolin (Al_2_O_3_(SiO_2_)_2_(H_2_O)_2_) was used as an inert ingredient to replace the yerba mate extract and maintain a constant nutrient concentration across experimental diets, as shown below.

Experimental treatments included one control diet (0% yerba mate extract and 4% of kaolin) and three diets with increasing inclusion levels of yerba mate extract (1% of extract and 3% of kaolin; 2% of extract and 2% of kaolin; and 4% of extract and 0% of kaolin), all expressed on a percentage of dry matter basis. The ingredient and chemical compositions of the diets are shown in Table 1.

The yerba mate extract was produced by Centro Flora (Botucatu, SP, Brazil), from fresh leaves of *Ilex paraguariensis* by water:ethanol 75:25 v/v extraction at 90 °C, which were freeze dried until they reached 91.8% dry matter. Total phenolic concentration was 21.7 eq-g gallic acid/100 g extract, and contained 6% caffeine. The extract used in this experiment was the same as the one used by Zawadzki et al. [26], in which it is reported a complete characterization of the phenolic compounds using an ultra-performance liquid chromatography electrospray ionization mass spectrometry analysis. Bromatological characterization of the dried extract was performed and the extract had a composition of 10% ash, 10.38% crude protein, 0.13% ether extract, 0.71% neutral detergent fiber, 0.68% acid detergent fiber, 0.18% lignin, and 4.15 Mcal/kg of gross energy. The extract was mixed with the concentrate and at feeding time the concentrate was mixed into the silage.

Upon animals’ arrival, the control diet (0% of yerba mate extract) was provided for all animals for three days, at the transition from third to fourth day, measurements and sample collections were made for blood baseline parameters. On the fourth day of the experiment, the animals started to be fed the previously assigned experimental diets for a total of 50 days (until time of slaughter).

For the whole experiment (53 days), before morning feeding, feeders were emptied and refusals were weighed to adjust the diet to contain at least 10% daily refusal. Approximately 10% of daily refusals were sampled and stored in an identified plastic bag in a −20 °C freezer, weekly composite refusal samples were generated, and dry matter analysis was carried out to calculate dry matter intake (DMI).

Dietary ingredients were analyzed according to Association of Official Analytical Chemists [28] methods for dry matter (DM, method 934.01), ash (ASH, method 923.03), and ether extract (EE, method 920.85), and nitrogen (method 920.87), which was analyzed by the Kjeldahl method, this value was multiplied by 6.25 to obtain crude protein (CP). Neutral detergent fiber (NDF) was analyzed according to Mertens [29] using thermostable-α-amylase without sodium sulfate, and acid detergent fiber (ADF) and lignin (ADL) (method 973.18) were measured according to Van Soest et al. [30]. Gross energy (GE) was measured with a bomb calorimeter (C200 System, IKA, Staufen, Germany). Organic matter (OM) was calculated by subtracting the ash content from 100.

### 2.3. Blood Collection and Analysis

Sample collections were carried out on the 3rd and 49th days of the experiment, 2 h after morning feeding. The first sample was collected when all animals were fed the control diet to represent the baseline measurement, and the second collection was performed at the end of the experiment for measuring the changes in the blood count and biochemical parameters induced by dietary treatments. Blood was collected from the jugular vein using a Vacutainer^®^ (Franklin Lakes, NJ, USA) collection tube. In total, three tubes were collected from each animal, the first with sodium fluoride for hematological parameters analysis, the second with Ethylenediaminetetraacetic acid (EDTA) and the last one without any anticoagulants, both for biochemical analysis.

Blood cell counting was carried out immediately after collection with a BC-2800 Vet Mindray^®^ automatic counter (Nanshan, Shenzhen, China). Cell counting provided the hematocrit (HT, %) and the red blood cell distribution width (RDW, %), as well as the amount of hemoglobin (HB, g/dL) and the number of blood platelets (PT, 10^3^/µL), red blood cells (RBC, 10^6^/µL), and the total and different types of white blood cells (WBC, 10^3^/µL), such as neutrophils, lymphocytes, monocytes, eosinophils and basophils. Then, the mean corpuscular volume (MCV) was calculated as (HT/RBC) × 10; the mean corpuscular hemoglobin (MCH) was calculated as (HB/RBC) × 10; the mean corpuscular hemoglobin concentration (MCHC) was calculated as (HB/HT) × 10.

The biochemical analysis tubes were processed immediately after collection of the blood by centrifugation at 2000× *g* for 15 min, and the plasma was stored in identified 2 mL tubes in a freezer (−20 °C). Biochemical analyzer RX Daytona and commercial kits both manufactured by Randox^®^ (Crumlin, UK) were used to determine the following biochemical parameters: glucose (GLU) (code: GL 3815), triglycerides (TG) (code: TR 3823), total cholesterol (CHOL) (code: CH 3810), high density lipoprotein (HDL-C) (code: CH 3811), blood urea nitrogen (BUN) (code: UR 3825), creatinine (CRE) (code: CR 3814), albumin (ALB) (code: AB 3800), total protein (TP) (code: TP 4001), creatinine kinase (CK) (code: CK 3812), gamma-glutamyl transferase (GGT) (code: GT 3817), and aspartate aminotransferase (AST) (code: AS 3804). Low-density lipoprotein (LDL-C) was calculated as (CHOL) - (HDL-C) - (TG/5); very-low-density lipoprotein (VLDL-C) was calculated as (TG/5); globulin (GLB) was calculated as (TP − ALB); the ALB:GLB ratio was calculated as (ALB/GLB); the BUN:CRE ratio was calculated as (BUN/CRE); the atherogenic index (AI) was calculated as [(CHOL - (HDL-C))/(HDL-C)].

### 2.4. Performance Control and In Vivo Biometrics

The weights of the animals were recorded every 14 days using an electronic scale. After measuring dry matter intake (DMI) and daily weight gain (DWG), the feed conversion (FC = DMI/DWG) and efficiency (FE = DWG/DMI) were calculated. On the last weighing procedure of the experiment, the day before slaughtering, the final live weight (FLW) and body dimensions of the animals were measured. The body condition score (BCS) was measured according to Russel [31] by palpation of the posterior dorsum of the animal and visual assessment by assigning scores ranging from 1 (very thin) to 5 (very fat). The length (LH), height of withers (HW), height of rump (HR), and girth (GH) were measured according to Pinheiro and Jorge [32]. The body compactness index (BCI) was calculated as FW/LH.

### 2.5. Slaughter and Carcass Biometrics

The animals were fed for 53 days to obtain live weights close to 40 kg. Before slaughter, the animals underwent 16 h of fasting of feed, and weighed again at the end of the fasting. This measurement was denoted as the body weight at slaughter (BWS). Slaughter was carried out at the experimental slaughterhouse at FZEA-USP by trained employees following standard procedures. Following proper stunning and exsanguination, the skin, head and hooves were removed from the carcasses, and eviscerations were made. After those steps, the hot carcass weight (HCW) was measured.

After measuring the HCW, carcass dimensions were measured before taking them to cold storage. The measurements were carried out according to Sañudo and Sierra [33] and Carrasco et al. [34], and the outer carcass length (OCL), inner carcass length (ICL), chest girth (CG), chest depth (CD), hind limb width (HLW), hind limb girth (HG), leg width (LW), and pelvic limb length (LL) were measured. The following indexes were calculated: carcass compactness index (CCI), which was calculated as CCW/ICL, and leg compactness index (LCI), which was calculated as LW/LL.

Carcasses were then placed into cold storage (2 °C) for approximately 24 h. On the following day, carcasses were weighed, and the measurements were denoted as the cold carcass weight (CCW). The carcasses were split into two, and the half carcasses were divided into forequarters and hindquarters. The carcasses were cut between the 12th and 13th ribs, and the fat thickness (FT) was measured using a caliper ruler. The ribeye area (RA) was measured from the same carcass piece by image analysis using ImageJ 1.8.0 software (Bethesda, MD, USA).

Carcass yield was calculated from the weight measurements of the animal body and carcass: hot carcass yield (HCY) was calculated as (HCW/BWS) × 100, cold carcass yield (CCY) was calculated as (CCW/BWS) × 100, carcass yield (CY) was calculated as (CCW/FW) × 100, and chilling carcass weight loss (CCWL) was calculated as 100 − ((CCW/HCW) × 100).

### 2.6. Statistical Analysis

The experiment was carried out in a completely randomized block design. All procedures were analyzed by the MIXED procedure of Statistical Analysis Software (Cary, NC, USA). First, evaluation of the normality of the residuals and influencer points was carried out, and then the statistical analyses were performed. The model for animal performance variables was composed of fixed effects (treatment and block) and error. For blood variables, the model was composed of fixed effects (treatment and block), the initial values observed for each parameter as covariates (when *p* > 0.05, the covariates were removed from the model), and error. Orthogonal contrasts (linear, quadratic, and cubic) were performed using the coefficients from IML procedure to adjust unequal spaced treatments and was considered significant when *p* ≤ 0.05 and considered a tendency when 0.05 < *p* ≤ 0.10.

## 3. Results

The cubic effects of the orthogonal contrast for most of the evaluated parameters had no statistical significance (data not shown in table), when an effect was observed, this is noted in the text.

### 3.1. Health Parameters

The results from blood cell count analysis are reported in Table 2. Most of the parameters were not affected by treatments, except for the leukocyte cells. The absolute count of WBCs increased from 7.00 × 10^3^ cells/µL in the control to an average of 7.97 × 10^3^ cell /µL (data not shown) in the treated animals, and the contrast analysis indicated a cubic effect (*p* = 0.05). The increased WBC count is explained by the increase in lymphocytes with a cubic effect (*p* = 0.04) and the segmented neutrophils (*p* = 0.02) with a quadratic effect.

The effects of increasing yerba mate extract on serum biochemical parameters are presented in Table 3. Animals had a linear increase in the total protein of the blood serum (*p* = 0.04) from 5.76 g/dL in the control to 5.99 g/dL in the treatment with the greatest level of yerba mate extract. This effect on total protein is caused by the increased amount of globulin (*p* = 0.05), and it also affected the albumin:globulin ratio (quadratic effect, *p* = 0.04). Regarding the lipid fraction of the blood, triglycerides (*p* = 0.03) and VLDL (*p* = 0.03) experienced a quadratic effect by the treatment, showing a reduction in triglycerides and VLDL in the blood of animals fed up to 2% yerba mate extract; however, no difference in the other constituents of the blood lipid parameters (cholesterol, HDL, LDL, and AI) were observed.

Creatinine kinase was used to investigate the integrity of the skeletal and cardiac muscle and had a tendency to have a cubic effect (*p* = 0.07). The liver function marker GGT showed a linear increase (*p* < 0.01) representing a 23.84% increase in the amount of the enzyme between the control and the greatest level of extract inclusion. Kidney function markers also had a linear increase in creatinine (*p* = 0.03) and a tendency for a linear reduction in the urea:creatinine ratio (*p* = 0.06).

### 3.2. Animal Performance

The animal performance results are presented in Table 4. The average initial live weight of the animal was 23.88 ± 0.22 kg, and the initial live weight from each treatment had no difference. The final live weight tended to have a quadratic response (*p* = 0.09), and animals fed up to 2% yerba mate extract had an increased final weight of approximately 2% compared to the control group. In contrast, animals fed the greatest level had a strong reduction in final live weight (approximately 8.87% compared to the control group and 10.64% compared to treatment with 2% extract inclusion). The body compactness index had a quadratic effect (*p* = 0.04). A linear negative effect was observed for total weight gain (*p* = 0.04), daily weight gain (*p* = 0.04), dry matter intake (*p* = 0.03), dry matter intake per live weight (*p* = 0.02), and live weight loss after 16 h of fasting (*p* = 0.04).

### 3.3. Carcass Metrics and Yield

The carcass yield and metrics are presented in Table 5; the parameters of hot carcass weight (*p* = 0.034), cold carcass weight (*p* = 0.03), cold carcass yield (*p* = 0.01), and carcass yield (*p* < 0.01) had a cubic response to treatments. Quadratic tendencies caused by the treatments were observed for the hot carcass yield (*p* = 0.07). Regarding the carcass metrics, the results show a linear treatment effect for the outer carcass length (*p* = 0.01) and quadratic effect differences for hind limb width (*p* = 0.07), hind limb girth (*p* < 0.01), and leg width (*p* = 0.01). Index parameters of the carcass had a cubic effect for the carcass compactness index (*p* = 0.01) and a quadratic effect for the leg compactness index (*p* = 0.01). Fat thickness (*p* = 0.01) and ribeye area (*p* = 0.06) had linear and quadratic effects, respectively.

## 4. Discussion

### 4.1. Health Parameters

This study tested increasing levels of yerba mate extract into the diet of growing lambs and its effects on the blood profile and animal performance. The blood count and biochemical parameters are good indicators to help to understand the overall health status of the animals. For the blood count analysis, a reference range was used as the basis for the interpretation of the results because cell count parameters have consistent data. On the other hand, no reference interval parameter of the blood biochemistry was used. According to Braun et al. [35], a great variation can be observed for these values, and usually the literature that has this information lacks consistency in animal models, analytical and statistical methods.

To begin a global analysis and to evaluate a new feed additive, it is important to first evaluate the health status of the animals. In general, all blood count parameters were in the normal range compared to that in the literature [36], suggesting that the general health status of the animals remained normal, despite the dietary treatment. Most of the blood count parameters were not affected by the treatment, except for some WBC parameters. The same response was observed by Beigh et al. [37] in their study with lambs fed a cocktail of exogenous fibrolytic enzymes and/or *Artemisia absinthium* L. (known as wormwood herb), and by Morsy et al. [38], who used red propolis extract with antioxidant characteristics to feed Santa Ines ewes; their results showed that the extract or even the plant itself in the diet increased the number of leukocyte counts.

White blood cell is a sensitive component to oxidative stress; during a period of stress (e.g., weaning), body increases cortisol production, which inhibits synthesis and stimulate breakdown of protein due to its oxidative activity [39]. The defense system is dependent on immunoglobulins, which are reduced by elevated levels of cortisol; another impact of this hormone is the atrophy of the lymphoid tissue [40], consequently, a reduction in the leukocytes count is observed [41,42]. Based on this, we could assume that the antioxidants in the extract affects the oxidative process caused by the stress and an increased number of white blood cells and globulins were observed.

Increasing WBC count occurred due to the increased number of segmented neutrophils and lymphocytes. The WBC system is one of the most important components of the body’s defense system. Neutrophils are cells that are considered the first barrier against health problems associated with microorganism infection, as well as with tissue trauma and inflammatory signals [43]. Usually, those cells are recruited from the blood to the site where an infection or even a risk of infection is detected [44].

Lymphocytes are the main WBC component (in quantity) in the blood. The three types of cells (T, B, and natural killer cells) are crucial to the immune system, and their function is recognized as producers of antibodies and killer cells that could damage the organism [45]. These components of WBCs play a major role in body surface protection, especially in the enteric system, mainly protecting mucosa against the proliferation of viral infections [46].

According to Eckersall [47], total protein is divided into two subclasses. Firstly, albumin, which is the main protein in the serum composition and is an important regulator of colloid osmotic pressure and blood volume. Albumin is also an important marker for nutritional protein status because it is largely used in muscle protein turnover. Secondly, globulin, which is a class of proteins that includes different types of proteins, such as proteins produced by the immune system and liver.

The results of our study show an increase in the levels of total blood protein and an increase in globulins in animals fed up to 2% yerba mate extract. Morsy et al. [38] presented similar results showing that their red propolis extract increased the globulin concentration, which indicates that isoflavonoids found in the extract could activate the immune system of the ewes. Also, the extract used in our work is an important source of caffeine, which is classified as an alkaloid substance, and Chaturvedi et al. [48], showed in in vitro cell models that alkaloids components could have immunomodulatory activity. These results support the hypothesis that bioactive compounds present in the yerba mate extract could also have an effect on the immune systems of growing lambs.

Reductions in some components of the lipid composition of the blood (such as triglycerides and VLDL) of lambs could be explained by inhibition of the synthesis of cholesterol in the liver by flavonoids and phenolic acids [49,50,51]; however, in our study, inhibition occurred only for VLDL and did not change LDL.

A sensitive blood marker for muscle damage was used. According to Houpert et al. [52], creatinine kinase (CK) has a high concentration in the muscle (approximately 4000–5000 U/g) compared to that in the plasma (50–100 U/g), and minimal damage could rapidly increase the concentration in the blood. Our results showed no trends, and two peaks for blood CK were observed in the animals fed 1% and 4% yerba mate extract. Those peaks were out of the normal range; thus, it is difficult to explain the reason for that.

Gamma-glutamyltransferase (GGT) is one of the markers for liver damage, and enzymes in the plasma range from 20–52 U/dL [53]. Increasing plasma levels have been reported in different studies and are associated with hepatobiliary disorders in sheep, such as fasciolosis [54,55], facial eczema [56], and even liver fibrosis due to long-term ingestion of *Tephrosia cinerea* [53]. However, to characterize liver damage, other biochemical parameters of the blood need to be evaluated, such as albumin concentration and aspartate aminotransferase (AST), and both of those parameters did not change when the treatments were applied.

### 4.2. Animal Performance and Carcass Metrics

Animal performance data presented in this study showed positive results and is similar to results reported in the literature using Dorper breed and its cross-breeds [57,58,59]. The effect of high levels of yerba mate extract (4%) resulted in a reduction in dry matter intake and, consequently, a reduction in the general performance of the lambs; on the other hand, inclusions of up to 2% caused an increase in dry matter intake, total weight gain, and daily weight gain.

The results of this study agree with the observations made by Po et al. [24], where they fed Dorper ewes 2.5% yerba mate dried leaves, and their results showed a reduction in the feed intake of the ewes a few weeks postpartum, indicating that 2.5% of dried leaves may exceed the optimum point of inclusion, in our case, inclusion greater than 2% caused a reduction in the performance of the lambs. The yerba mate extract used is rich in hydrolysable tannins and alkaloids, as caffeine [26], and those compounds have both beneficial and adverse effects on ruminant nutrition. According to the literature, inclusion of tannins at moderated levels in ruminants’ diets could increase ruminal undegraded protein [60] and inclusion of caffeine also in moderate levels could have beneficial effects on the central nervous system of the animal [61]. However, both components have a bitter taste, and consequently influence the palatability of the diet and animal production if included at high levels [60,61].

Although, even with a reduction in the production parameters at the greatest level of inclusion of extract, the index of feed and efficiency conversion did not change, showing that the feed intake had a similar rate of reduction as that of weight gain.

In our study, increasing levels of yerba mate extract in the diet decreases fat thickness. A recent study using mice as models showed that yerba mate could decrease the differentiation of preadipocytes and reduce the accumulation of lipids into those cells [62]. This occurs due to the adipogenesis modulatory characteristic of yerba-mate, causing regulation of the gene expression levels of pro-adipogenic transcription factors [63,64,65].

Up to 2% of inclusion of yerba mate extract caused an increased ribeye area, that parameter is used to estimate the muscle production of the body. The relationship between thickness and ribeye area are an important indicator of how fatty or lean the meat is. In this context, animals fed up to 2% of yerba mate extract had a greater amount of lean meat and a reduced fat deposition. According to Li et al. [66], lean red meat is lower in saturated fat, which has been recently recommended for a healthy human diet [67].

From the other carcass parameters, we observed a similar response with increase in productive parameters (up to 2% of extract inclusion); however, a strong decrease was observed when high levels (4%) of yerba mate extract were used. The carcass metrics also had a reduction in the carcass and leg compactness indexes at the highest treatment level, which indicates that high levels of added yerba mate extract negatively affected the productive parameters of the lambs, probably due to a bitter taste of the extract as presented above.

## 5. Conclusions

In general, the addition of yerba mate extract to the diet does not change the nutritional status of the animals; however, inclusion of yerba mate extract up to 2% of the dry matter intake increases the levels of leukocyte cells and globulins in the serum, and reduces VLDL and triglycerides. Such responses are desired in lamb production and can lead to improvement in the health and production of the animals.

Additionally, productive parameters and carcass yield is improved in animals fed up to 2% yerba mate extract; leading to a greater production of a lean tissue, which is recommended for a healthy human diet and desired in the meat industry. However, inclusion beyond 2% causes reductions in animal performance and carcass characteristics, which it is not desirable, and demonstrates the limitation of yerba mate utilization.

## Figures and Tables

**Table 1:** Ingredient and chemical composition of experimental diets in a dry matter basis.

Ingredients, g/kg	Treatments			
	0%	1%	2%	4%
Corn silage	400	400	400	400
Ground corn	330	330	330	330
Soybean meal	205	205	205	205
Salt	1.5	1.5	1.5	1.5
Dicalcic phosphate	1.5	1.5	1.5	1.5
Minerals ^1^	22	22	22	22
Yerba mate extract	-	10	20	40
Kaolin	40	30	20	-
**Chemical Composition, g/kg otherwise stated**				
Dry matter	689	688	688	686
Organic matter	896	904	913	930
Crude protein	215	216	217	219
Ether extract	20.9	20.9	20.9	20.9
ADF	182	182	182	182
aNDF	345	345	345	346
Lignin	44.8	44.8	44.8	44.8
Gross energy, MJ/kg DM	4.19	4.23	4.27	4.35

^1^ Mineral composition per kilogram: calcium (maximum)-218 g; calcium (minimum)-190 g; cobalt (min)-148 mg; copper (min)-2664 mg; sulfur (min)-64 g; fluoride (max)-1600 mg; phosphorus (min)-160 g; iodine (min)-141 mg; manganese (min)-2220 mg; selenium (min)-37 mg; and zinc (min)-7992 mg.

**Table 2:** Blood cell count of growing lambs fed increased levels of yerba mate extract.

Cell Count ^1^	Treatments					*p* ^3^		
	0%	1%	2%	4%	MSE ^2^	Treat	Lin	Quad
Erythrogram								
RBC, 10^6^/µL	14.4	14.4	14.0	14.3	0.28	0.66	0.67	0.39
HB, g/dL	14.1	13.9	13.4	13.8	0.30	0.50	0.41	0.28
HT, %	41.8	40.8	39.7	40.6	0.75	0.33	0.29	0.14
MCV, fL	28.8	28.0	28.5	28.6	0.31	0.33	0.89	0.24
MCH, pg	9.63	9.61	9.57	9.53	0.14	0.95	0.57	0.97
MCHC, %	33.6	34.1	33.6	33.9	0.38	0.74	0.88	0.89
RDW, %	18.2	18.2	18.0	18.2	0.24	0.88	0.92	0.61
Leukogram								
WBC, 10^3^/µL	7.00	8.25	7.99	8.41	0.25	<0.01	<0.01	0.07
Absolute count, 10^3^/µL								
Band neutrophils	0.028	0.018	0.000	0.032	0.014	0.38	0.79	0.11
Segmented neutrophils	2.02	3.39	3.40	3.18	0.36	0.03	0.08	0.02
Lymphocytes	4.04	5.15	4.44	4.48	0.31	0.11	0.80	0.18
Monocytes	0.024	0.108	0.076	0.076	0.042	0.56	0.60	0.35
Eosinophils	0.075	0.068	0.091	0.084	0.026	0.93	0.71	0.87
Basophils	0.025	0.019	0.024	0.020	0.012	0.98	0.81	0.97
Thrombogram								
PT, 10^3^/µL	397	533	549	541	46.3	0.09	0.07	0.09

^1^ RBC—red blood cells; HB—hemoglobin; HT—hematocrit; MCV—mean corpuscular volume; MCH —mean corpuscular hemoglobin; MCHC—mean corpuscular hemoglobin concentration; RDW—red blood cells distribution width; WBC—white blood cells; PT—platelets; ^2^ MSE—mean square error; ^3^ *p* values from treatment (Treat) and orthogonal contrast (linear and quadratic) are significantly different if *p* ≤ 0.05 and are a tendency if 0.05 < *p* < 0.10.

**Table 3:** Blood biochemical parameters of growing lambs fed increased levels of yerba mate extract.

Parameter ^1^	Treatments					*p* ^3^		
	0%	1%	2%	4%	MSE ^2^	Treat	Lin	Quad
TP, g/dL	5.76	5.93	6.05	5.99	0.07	0.03	0.03	0.04
ALB, g/dL	3.55	3.53	3.56	3.52	0.06	0.95	0.76	0.76
GLB, g/dL	2.25	2.31	2.44	2.42	0.03	<0.01	<0.01	0.05
A:G	1.60	1.56	1.47	1.49	0.02	<0.01	<0.01	0.04
GLU, mg/dL	83.7	82.0	85.0	83.0	1.58	0.58	0.99	0.72
TG, mg/dL	35.1	32.9	31.0	39.1	2.15	0.08	0.14	0.03
CHOL, mg/dL	44.9	47.5	46.2	46.5	1.91	0.79	0.74	0.59
HDL-C, mg/dL	18.0	19.8	20.0	20.0	1.15	0.52	0.39	0.33
LDL-C, mg/dL	18.2	23.1	20.3	18.4	1.89	0.23	0.64	0.14
VLDL-C, mg/dL	7.02	6.58	6.20	7.83	0.43	0.08	0.14	0.03
AI	1.45	1.41	1.34	1.43	0.14	0.95	0.97	0.59
CK, U/dL	90.2	110.3	86.9	125.6	10.13	0.04	0.04	0.33
AST, U/dL	82.0	73.5	75.8	71.2	4.27	0.34	0.14	0.58
GGT, U/dL	58.2	60.6	64.1	72.1	2.02	<0.001	<0.01	0.63
BUN, mg/dL	43.7	45.8	47.3	44.6	2.31	0.71	0.84	0.27
CRE, mg/dL	0.84	0.80	0.99	0.99	0.06	0.05	0.03	0.71
BUN:C	53.1	61.2	47.6	43.2	5.09	0.09	0.06	0.55

^1^ TP—total protein; ALB—albumin; GLB—globulin; A:G—ratio albumin:globulin; GLC—glucose; TG —triglicerides; CHOL—cholesterol; HDL-C—high density lipoprotein; LDL-C—low density lipoprotein; VLDL—very low density lipoprotein; AI—atherogenic index; CK—creatinin kinase; AST —aspartate aminotrasferase; GGT—gamma-glutamyl transpeptidase; BUN—blood urea nitrogen; CRE—creatinine; BUN:C—ratio of blood urea nitrogen:creatinine; ^2^ MSE—mean square error; ^3^ *p* from treatment (Treat) and orthogonal contrast (linear and quadratic) are significantly different if *p* ≤ 0.05 and are a tendency if 0.05 < *p* < 0.10.

**Table 4:** Performance parameters and body morphometry of growing lambs fed increased levels of yerba mate extract.

Parameter ^1^	Treatments					*p* ^3^		
	0%	1%	2%	4%	MSE ^2^	Treat	Lin	Quad
Performance								
ILW, kg	24.2	24.0	24.2	23.6	0.22	0.24	0.09	0.47
FLW, kg	39.9	39.1	40.8	36.4	0.93	0.02	0.02	0.09
TWG, kg	15.8	15.1	16.6	12.8	0.89	0.04	0.04	0.11
DWG, g/d	267	256	282	217	15.2	0.04	0.04	0.11
DMI, g/d	1153	1152	1171	1002	49.3	0.07	0.03	0.16
DMI LW, %	3.61	3.67	3.60	3.28	0.11	0.06	0.02	0.19
FC	3.79	4.05	3.76	4.00	0.14	0.38	0.52	0.87
EF	0.26	0.25	0.27	0.26	0.01	0.42	0.85	0.90
In vivo measurements								
LWLF, %	4.76	5.22	4.35	3.93	0.29	0.03	0.01	0.45
BCE, cm	3.67	3.83	4.00	3.89	0.17	0.57	0.36	0.29
LH, cm	65.0	64.7	64.4	65.6	1.0	0.87	0.65	0.50
GH, cm	78.6	79.1	78.1	76.9	1.2	0.62	0.23	0.69
HW, cm	56.7	57.7	57.2	56.8	0.7	0.75	0.82	0.40
HR, cm	59.4	61.1	59.7	59.6	0.9	0.57	0.74	0.48
BCI, kg cm^−1^	0.599	0.601	0.618	0.559	0.013	0.03	0.04	0.04

^1^ ILW—initial live weight; FLW—final live weight; TWG—total weight gain; DWG—daily weight gain; DMI—dry matter intake; DMI LW—percentage of dry matter intake related to the average of weight; FC—feed conversion; EC—efficiency conversion; LWLF—live weight loss after 16 h of fasting; BCE—body condition score; LH—length; GH—girth; HW—height of withers; HR—height of rump; BCI—body compactness index; ^2^ MSE—mean square error; ^3^ *p* from treatment (Treat) and orthogonal contrast (linear and quadratic) are significantly different if *p* ≤ 0.05 and are a tendency if 0.05 < *p* < 0.10.

**Table 5:** Carcass traits of growing lambs fed increased levels of yerba mate extract.

Parameter ^1^	Treatments					*p* ^3^		
	0%	1%	2%	4%	MSE ^2^	Treat	Lin	Quad
Carcass yield								
HCW, kg	17.7	17.3	19.0	17.0	0.48	0.04	0.45	0.06
HCY, %	47.3	47.0	49.7	48.1	0.57	0.02	0.13	0.07
CCW, kg	17.3	16.9	18.6	16.6	0.48	0.03	0.46	0.06
CCY, %	46.2	46.0	48.6	47.0	0.55	<0.01	0.12	0.05
CY, %	44.0	43.6	46.5	45.2	0.55	<0.01	0.04	0.08
CCWL, %	2.36	2.30	2.06	2.33	0.10	0.18	0.77	0.06
Carcass measurements								
OCL, cm	59.4	59.6	59.0	57.6	0.54	0.05	0.01	0.36
ICL, cm	53.1	53.4	52.0	51.8	0.70	0.27	0.10	0.92
CG, cm	66.6	65.6	67.2	66.9	1.10	0.74	0.61	0.98
CD, cm	25.1	25.4	25.4	25.8	0.38	0.55	0.17	0.87
HLW, cm	19.6	19.9	20.6	20.1	0.32	0.16	0.26	0.07
HG, cm	57.2	58.6	60.8	57.8	0.59	<0.01	0.54	<0.01
LW, cm	15.8	15.9	16.3	15.3	0.21	0.02	0.08	0.01
LL, cm	30.0	30.1	29.1	30.0	0.42	0.33	0.81	0.22
CCI, kg cm^−1^	0.32	0.32	0.36	0.31	0.008	<0.001	0.61	<0.01
LCI, kg cm^−1^	0.65	0.66	0.71	0.67	0.01	0.03	0.26	0.01
FT, mm	2.75	2.55	2.21	1.82	0.40	0.41	0.10	0.91
RA, mm ^2^	1330	1301	1519	1215	79	0.09	0.41	0.06

^1^ HCW—hot carcass weight; HCY—hot carcass yield; CCW—cold carcass weight; CCY—cold carcass yield; CY—carcass yield; CCWL—chilling carcass weight loss; OCL—outer carcass length; ICL—inner carcass length; CG—chest girth; CD—chest depth; HLW—hind limb width; HG—hind limb girth; LW—leg width; LL—pelvic limb length; CCI—carcass compactness index; LCI—leg compactness index; FT—fat thickness; RA—ribeye area; ^2^ MSE—mean square error; ^3^ P values from treatment (Treat) and orthogonal contrast (linear and quadratic) are significantly different if *p* ≤ 0.05 and are a tendency if 0.05 < *p* < 0.10.
